# Gene co-expression networks associated with carcass traits reveal new pathways for muscle and fat deposition in Nelore cattle

**DOI:** 10.1186/s12864-018-5345-y

**Published:** 2019-01-10

**Authors:** Bárbara Silva-Vignato, Luiz L. Coutinho, Mirele D. Poleti, Aline S. M. Cesar, Cristina T. Moncau, Luciana C. A. Regitano, Júlio C. C. Balieiro

**Affiliations:** 1 College of Agriculture “Luiz de Queiroz”, University of São Paulo, Piracicaba, SP 13418-900 Brazil; 2 College of Animal Science and Food Engineering, University of São Paulo, Pirassununga, SP 13635-900 Brazil; 3 Federal University of Lavras, Lavras, MG 37200-000 Brazil; 4 Embrapa Pecuária Sudeste, São Carlos, SP 13560-970 Brazil; 5 College of Veterinary Medicine and Animal Science, University of São Paulo, Pirassununga, SP 13635-900 Brazil

**Keywords:** Backfat thickness, Functional enrichment analysis, Ribeye area, RNA-Seq data, WGCNA

## Abstract

**Background:**

Positively correlated with carcass weight and animal growth, the ribeye area (REA) and the backfat thickness (BFT) are economic important carcass traits, which impact directly on producer’s payment. The selection of these traits has not been satisfactory since they are expressed later in the animal’s life and multigene regulated. So, next-generation technologies have been applied in this area to improve animal’s selection and better understand the molecular mechanisms involved in the development of these traits. Correlation network analysis, performed by tools like WGCNA (Weighted Correlation Network Analysis), has been used to explore gene-gene interactions and gene-phenotype correlations. Thus, this study aimed to identify putative candidate genes and metabolic pathways that regulate REA and BFT by constructing a gene co-expression network using WGCNA and RNA sequencing data, to better understand genetic and molecular variations behind these complex traits in Nelore cattle.

**Results:**

The gene co-expression network analysis, using WGCNA, were built using RNA-sequencing data normalized by transcript per million (TPM) from 43 Nelore steers. Forty-six gene clusters were constructed, between them, three were positively correlated (*p*-value< 0.1) to the BFT (Green Yellow, Ivory, and Light Yellow modules) and, one cluster was negatively correlated (*p*-value< 0.1) with REA (Salmon module). The enrichment analysis performed by DAVID and WebGestalt (FDR 5%) identified eight Gene Ontology (GO) terms and three KEGG pathways in the Green Yellow module, mostly associated with immune response and inflammatory mechanisms. The enrichment of the Salmon module demonstrated 19 GO terms and 21 KEGG pathways, related to muscle energy metabolism, lipid metabolism, muscle degradation, and oxidative stress diseases. The Ivory and Light yellow modules have not shown significant results in the enrichment analysis.

**Conclusion:**

With this study, we verified that inflammation and immune response pathways modulate the BFT trait. Energy and lipid metabolism pathways, highlighting fatty acid metabolism, were the central pathways associated with REA. Some genes, as *RSAD2, EIF2AK2, ACAT1*, and *ACSL1* were considered as putative candidate related to these traits. Altogether these results allow us to a better comprehension of the molecular mechanisms that lead to muscle and fat deposition in bovine.

**Electronic supplementary material:**

The online version of this article (10.1186/s12864-018-5345-y) contains supplementary material, which is available to authorized users.

## Background

Heavy carcass weight is critical for meat producers since it is used as the primary parameter for payment in the slaughterhouses [1]. Positively correlated with carcass weight and animal growth, the ribeye area (REA) can be used as an indicator of muscularity, prime cuts, and the edible mass of carcass. Inversely proportional to REA, the backfat thickness (BFT) is related to the percentage of fat in the carcass. BFT is essential to protect the carcass during cooling, avoiding problems such as cold shortening, drip loss and dark cutting [2–4].

Despite the economic relevance for producers and to affect the final weight of the animals, the selection of these traits has not been satisfactory, since their maximum potential is expressed later in animal’s life [1]. Therefore, a better understanding of the biological processes that regulate these characteristics could help to elucidate the mechanisms of genetic inheritance, and consequently will increase animal selection accuracy. In this context, next-generation sequencing technologies have revolutionized genome and transcriptome analysis of complex organisms, generating large datasets and allowing the identification of new genes, metabolic pathways and biological processes that influence the phenotype [5–7]. Moreover, novel approaches have been developed to more reliably analyze large and multivariate datasets by associating them with traits of interest. Correlation network analysis has been widely used to analyze large datasets as a promissory method from systems biology, able to represent the complexity of a cellular transcription network [8–10].

The Weighted Correlation Network Analysis (WGCNA) is a system biology method that is used to explore the correlation patterns among genes in transcriptomic studies, providing a unique insight into the structure and behavior of molecular interactions [8, 9]. The WGCNA describes and permits the visualization of networks derived from large datasets. WGCNA can be used to explore the structure of modules within a co-expression network, to measure the relationship between genes and modules (module membership), explore the relationship between modules, or even to rank genes or modules associated to the studied traits [8].

Several works utilized gene co-expression networks analysis, in particular, the WGCNA tool, to evaluate complex traits in different species such as mice [11], humans [12], pork [13, 14], lamb [10] and, cattle [15–18] demonstrating the correlation between genes and phenotype successfully.

Kong et al. [16] utilizing the WGCNA tool to understand the molecular differences between efficient and inefficient cattle concerning residual feed intake in a Hereford x Angus population, identified a significant module with 764 genes negatively correlated with the trait of interest. With the results, the authors could infer that efficient animals probably have an increased energy production and better absorption of food nutrients compared to inefficient ones. Sabino et al. [10] used the WGCNA to do a nutrigenomics investigation in lambs, considering diet and sex differences in muscle and liver tissue, revealing a sex-dependent dietary effect on the transcriptome of the studied animals. In previous work from our group, Oliveira et al. [18] employed WGCNA tool to do an integrative miRNA-mRNA (microRNA – messenger RNA) study associated with intramuscular fat deposition in Nelore cattle, revealing potential regulatory mechanisms of gene signaling networks involved in fat deposition in bovine.

The present study aimed to gain molecular insights into economic important carcass traits of Nelore cattle and to identify putative candidate genes and metabolic pathways that regulate REA and BFT, by constructing a gene co-expression network using WGCNA and RNA sequencing data. Modules correlated with REA and BFT were identified, and the genes within each significant module were extracted to perform functional enrichment analysis, permitting us to better understand gene interaction, biological processes, and the metabolic pathways behind these complex traits.

## Results

For this study, we used RNA-sequencing normalized data by transcript per million (TPM) from 14,529 genes of 43 animals with contrasting genomic estimated breeding values (GEBV). Table 1 shows animal identification, phenotypic values, GEBVs, number of raw reads, mapped reads and percentage of mapped reads. The heritability values for REA and BFT were h^2^ = 0.27 and h^2^ = 0.21, respectively [19]. Additional file 1: Table S1 shows the contrasting GEBV groups for both traits, demonstrating that mean values in the low group were statistically different (*p*-value< 0.001) from the high group for REA and BFT. For REA, mean GEBV were − 2.94 in the low group (standard deviation, SD = 0.59) and, 3.14 in the high group (SD = 0.56); for BFT, mean GEBV were − 0.91 (SD = 0.12) and 1.24 (SD = 0.28) in the low and high group, respectively. The correlation analysis between the phenotypic (REA mean and SD values = 59.75 ± 9.50 cm^2^; BFT mean and SD values = 7.00 ± 3.41 mm) and GEBV values both for REA and BFT showed a strong correlation, with *r* = 0.79 and *r* = 0.84 for REA and BFT respectively, justifying our selection using genomic values. On the other hand, the correlation analysis between the GEBV of REA *x* BFT demonstrated that these traits were independent of one another in this particular study, with *r* = − 0.06.

**Table 1:** Phenotypic and genomic values, number of raw-reads, number and percentage of mapped reads from the selected 43 Nelore cattle

Animal	REA (cm^2^)^a^	GEBV REA^b^	BFT (mm)^c^	GEBV BFT^d^	Raw reads^e^	Mapped reads^f^	%^g^
1	56.25	− 2.13	15.00	1.62	24.40	10.30	42.21
2	73.25	3.22	4.00	−0.79	11.81	5.49	46.49
3	67.50	1.39	7.00	−0.91	24.89	16.07	64.56
4	79.00	0.85	7.00	−1.17	11.75	5.86	49.87
5	69.25	0.5	7.00	−0.83	16.43	7.11	43.27
6	58.75	1.51	15.00	1.63	20.40	13.57	66.52
7	58.00	−0.98	7.00	−0.84	19.98	8.27	41.39
8	80.25	2.69	10.00	0.77	23.35	12.62	54.05
9	54.00	−2.76	9.00	−0.02	25.56	16.87	66.00
10	62.50	2.09	14.00	1.63	12.36	5.57	45.06
11	75.25	1.65	6.00	−0.85	18.01	11.88	65.96
12	48.50	−3.54	9.00	−0.06	17.83	8.13	45.60
13	72.00	4.71	6.00	0.07	9.75	6.68	68.51
14	68.75	2.79	10.00	0.93	23.87	17.84	74.74
15	65.50	−0.44	6.00	−0.79	14.20	12.77	89.93
16	73.25	2.92	15.00	1.19	25.60	12.77	49.88
17	75.00	1.36	12.00	1.00	13.85	9.24	66.71
18	58.75	−2.24	9.00	0.73	16.04	12.32	76.81
19	74.75	0.53	5.00	−0.79	13.76	7.09	51.53
20	59.75	−2.13	11.00	1.1	11.20	5.67	50.63
21	71.00	1.16	5.00	−0.88	16.98	11.51	67.79
22	58.75	−2.51	8.00	0.35	18.08	10.12	55.97
23	79.75	3.47	5.00	−1.06	17.10	11.42	66.78
24	62.00	0.29	4.00	−1.02	17.13	11.25	65.67
25	59.75	−0.1	11.00	0.91	19.37	9.15	47.24
26	55.75	−0.86	11.00	1.48	16.22	10.58	65.23
27	52.00	−2.92	8.00	0.49	12.47	5.38	43.14
28	56.75	1.92	9.00	1.37	12.30	6.48	52.68
29	54.00	−2.19	4.00	0.05	15.57	6.67	42.84
30	56.25	−2.67	4.00	−0.5	14.52	6.84	47.11
31	58.00	−1.4	2.50	−1.03	8.79	4.08	46.42
32	66.20	3.22	4.00	−0.37	21.97	10.13	46.11
33	47.75	−2.4	2.00	−0.75	20.65	9.49	45.96
34	50.75	−3.88	6.00	−0.1	18.21	9.35	51.35
35	42.50	−3.45	6.00	0.31	13.29	6.20	46.65
36	51.25	−2.79	5.00	0.09	19.93	9.59	48.12
37	64.50	2.85	4.00	−0.01	17.13	7.75	45.24
38	63.00	2.53	9.00	0.68	20.53	6.40	31.17
39	52.50	−3.95	8.00	0.6	11.31	5.26	46.51
40	54.25	−1.01	12.00	0.93	23.86	11.27	47.23
41	66.75	3.26	3.50	−0.58	25.92	12.83	49.50
42	65.50	2.9	7.50	0.32	13.94	6.41	45.98
43	47.50	−1.67	10.00	1.04	25.05	12.76	50.94
Mean	59.75	0.29	7.00	0.07	17.13	9.35	53.85
SD^h^	9.50	2.44	3.41	0.87			

^a^Ribeye area; ^b^genomic estimated breeding values for REA; ^c^Backfat thickness; ^d^genomic estimated breeding values for BFT; ^e^millions of raw reads; ^f^millions of mapped reads; ^g^percentage of paired-end mapped reads; ^h^Standard Deviation

**Fig. 1:**
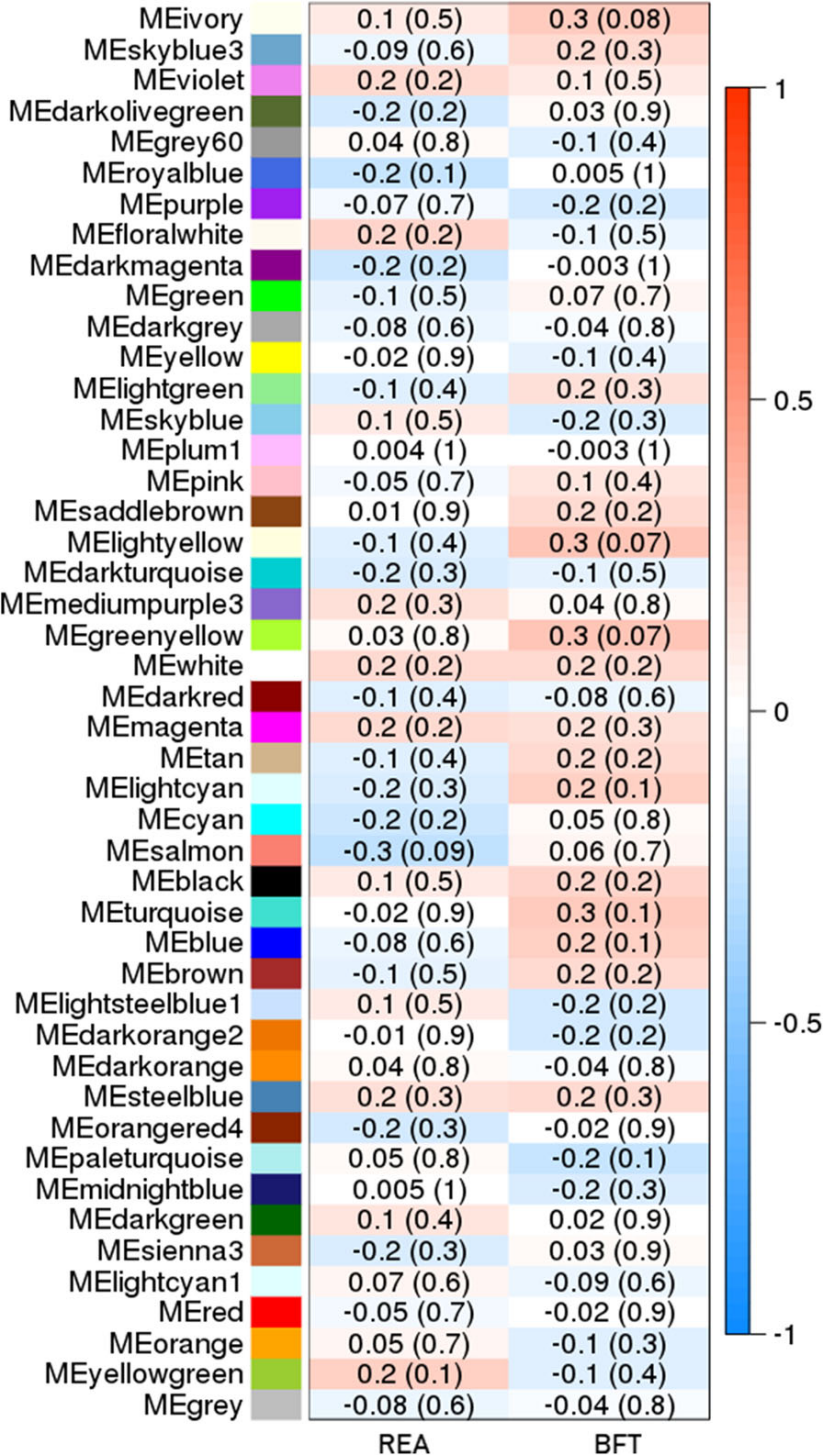
Module-trait associations between the module eigengenes (ME) and the studied traits, ribeye area (REA) and backfat thickness (BFT). Each row corresponds to a module eigengene, column to a trait. Each cell contains the Pearson’s correlation coefficients (numbers outside parentheses) and, the *p*-values of the correlation (numbers within parentheses). The graphic is color-coded by correlation according to the color legend, red represents a positive correlation and blue represents a negative one

**Fig. 2:**
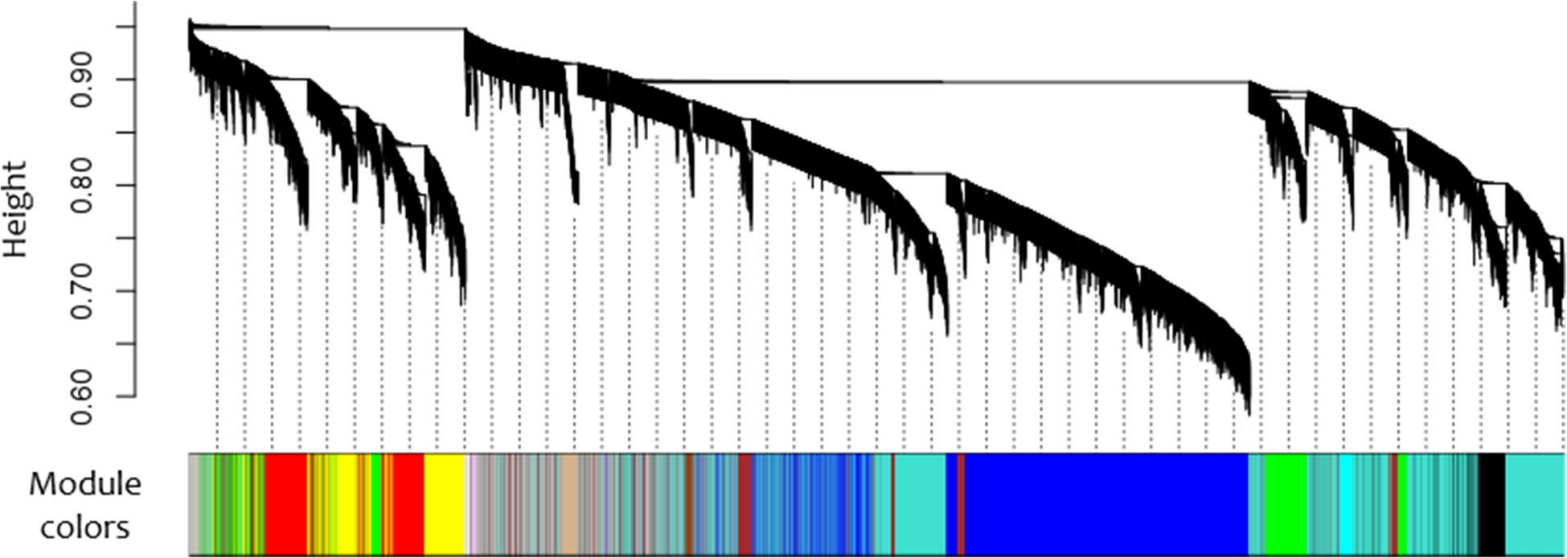
Cluster dendrogram of all genes from the selected Nelore steers. Cluster dendrogram of all genes, with dissimilarity based on topological overlap. The different colors in the bottom represent gene modules

The gene co-expression network analysis, performed using WGCNA program, resulted in the identification of 46 module eigengenes (ME) (Fig. 1). Figure 2 illustrates the hierarchical clustering tree (dendrogram) of all genes and modules colors and, in the supplementary material, there is a heatmap plot of the gene network (Additional file 2: Figure S1). Among the 46 identified ME, the Ivory, Light Yellow, Green Yellow, and Salmon modules showed a significant module-trait association (*p*-value< 0.1) with at least one of the studied phenotypes, demonstrating positive correlations of *r* = 0.3 with BFT (Ivory, Light Yellow and Green Yellow) and, a negative correlation of *r* = − 0.3 with REA (Salmon) (Fig. 1). The entire lists of genes in each of these modules were further analyzed using DAVID (Database for Annotation, Visualization and Integrated Discovery) version 6.8 and, WebGestalt 2017 (WEB-based GEne SeT AnaLysis Toolkit).

**Fig. 3:**
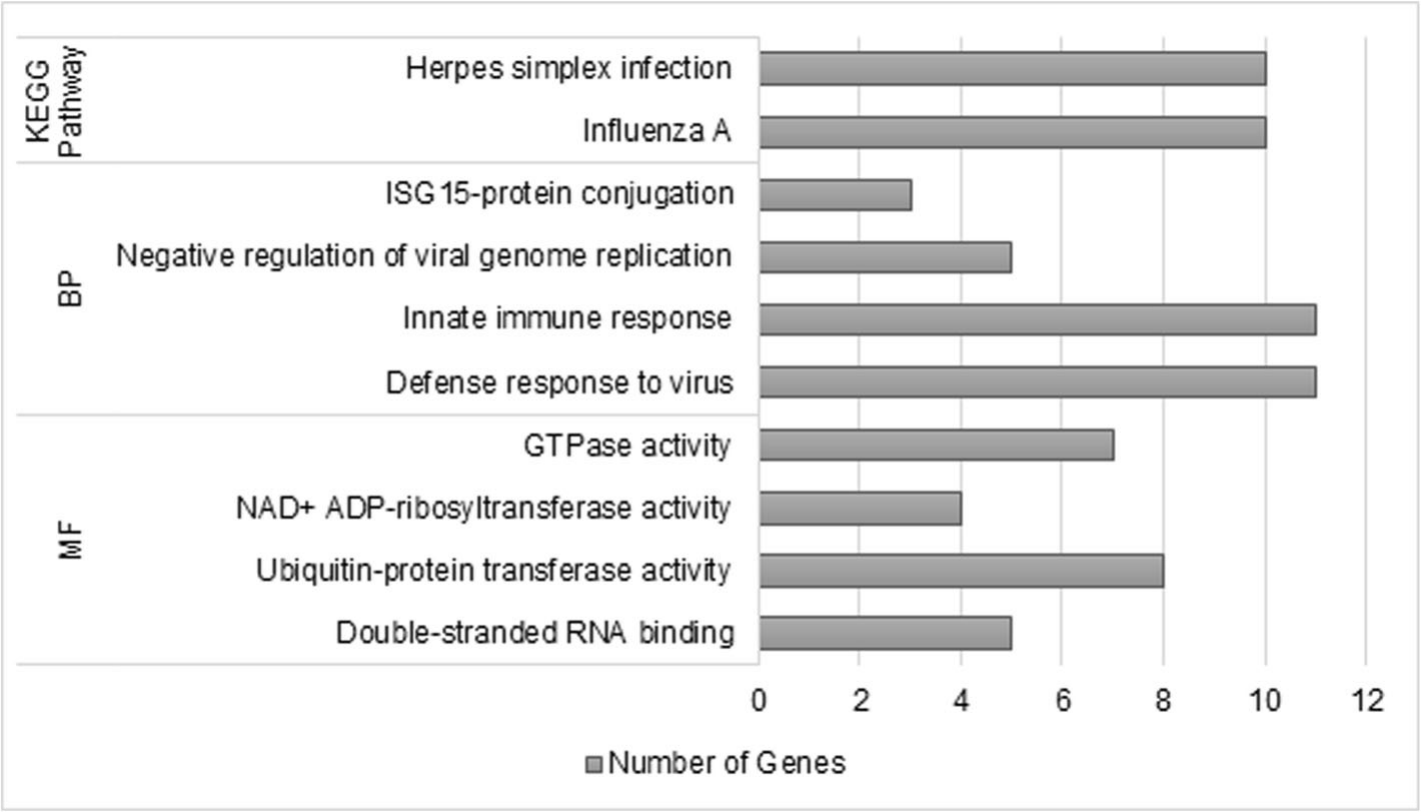
Functional enrichment analysis from the gene list of the Green Yellow module, performed by DAVID v6.8 (FDR < 0.05). Gene Ontology terms: BP – Biological Process; MF – Molecular Function

The Green Yellow module, positively correlated to BFT (*p*-value< 0.1), presented 146 co-expressed genes assigned for the enrichment analysis. Eight Gene Ontology (GO) terms divided into four Biological Processes (BP), four Molecular Functions (MF), and two KEGG (Kyoto Encyclopedia of Genes and Genomes) pathways were identified in the analysis performed using DAVID (FDR 5%) (Fig. 3, Additional file 3: Table S2).

The two KEGG pathways found were Influenza A (bta05164) and Herpes simplex infection (bta05168). The biological processes were defense response to virus (GO:0051607), innate immune response (GO:0045087), negative regulation of viral genome replication (GO:0045071) and, ISG15-protein conjugation (GO:0032020). The MF were double-stranded RNA binding (GO:0003725), ubiquitin-protein transferase activity (GO:0004842), NAD+ ADP-ribosyltransferase activity (GO:0003950) and, GTPase activity (GO:0003924). Most of them related to inflammation mechanisms and the immune system.

The functional enrichment analysis by WebGestalt revealed three KEGG pathways (Table 2), the different pathway identified in this analysis was the NOD-like receptor signaling pathway (bta04621), also related to immune response.

**Table 2:** KEGG Pathways (FDR < 0.05) identified by WebGestalt 2017 from the gene list of the Green Yellow module

KEGG Pathway ID^a^	Description	N Gene^b^	FDR^c^	Gene names
bta05168	Herpes simplex infection	9	4.77E-04	*PML, IFIT1, HLA-DMB, EIF2AK2, DDX58, IRF9, TAP1, OAS2, IFIH1*
bta05164	Influenza A	8	1.17E-03	*PML, HLA-DMB, EIF2AK2, DDX58, RSAD2, IRF9, OAS2, IFIH1*
bta04621	NOD-like receptor signaling pathway	6	4.40E-02	*CATHL5, IRF9, LOC511531, LOC512486, GBP5, OAS2*

^a^KEGG Pathway Identification (ID); ^b^Number of genes; ^c^Adjusted p-value for a false discovery rate (FDR) of 5%

**Fig. 4:**
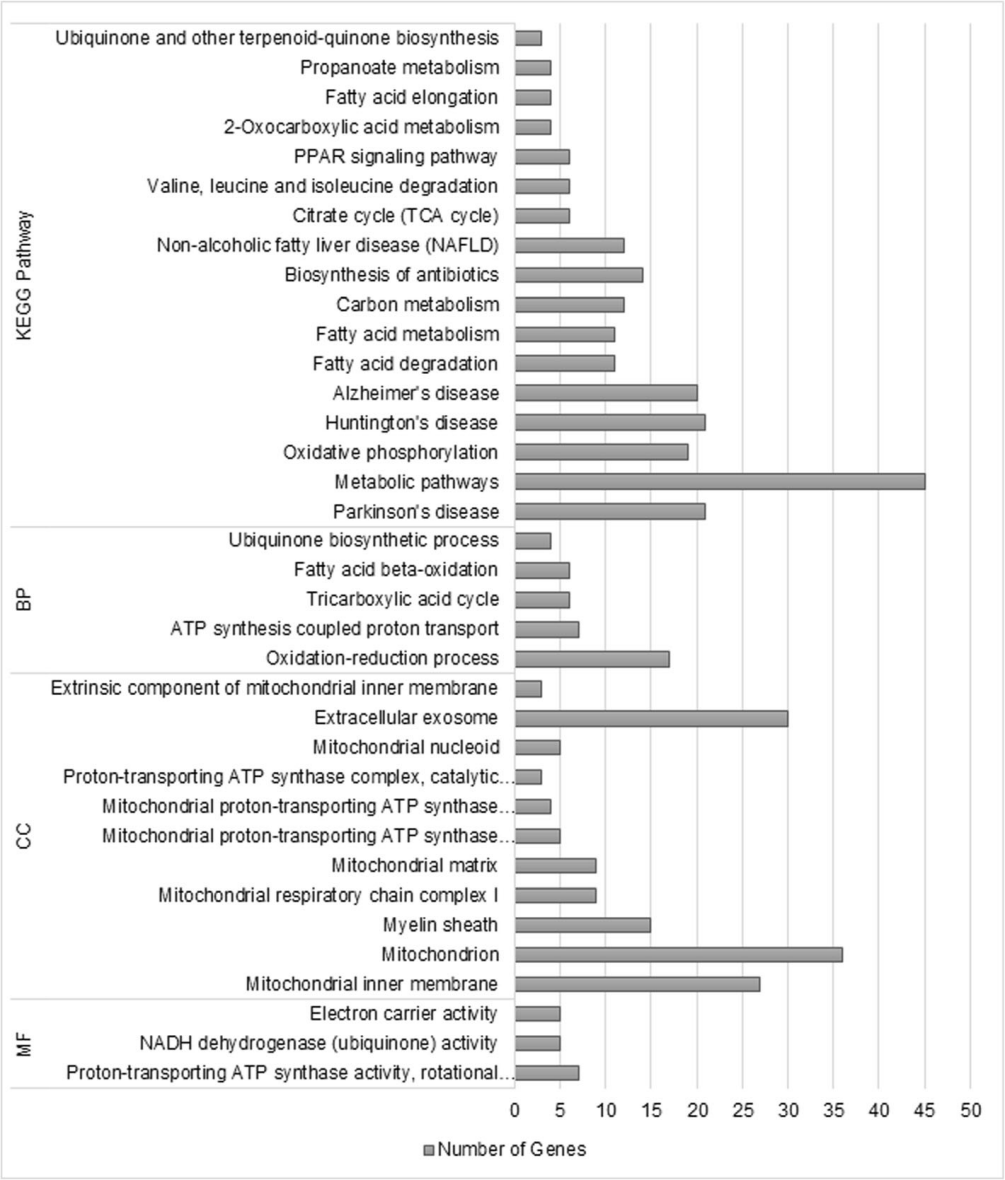
Functional enrichment analysis from the gene list of the Salmon module, performed by DAVID v6.8 (FDR < 0.05). Gene Ontology terms: BP – Biological Process; CC – Cellular Component; MF – Molecular Function

The Salmon module, negatively correlated with REA (*p*-value< 0.1), was constituted by 136 genes. Figure 4 and Additional file 4: Table S3 demonstrates the results of the functional enrichment analysis performed by DAVID (FDR 5%), we found five BP, eleven Cellular Components (CC) and, three MF terms.

The biological processes were mostly related to energy metabolism, like ATP synthesis coupled proton transport (GO:0015986), tricarboxylic acid cycle (GO:0006099) and, fatty acid beta-oxidation (GO:0006635). From the eleven CC identified, seven were mitochondrial constituents, such as mitochondrial inner membrane (GO:0005743), mitochondrial respiratory chain complex I (GO:0005747), mitochondrial matrix (GO:0005759) and, mitochondrial proton-transporting ATP synthase complex (GO:0005753). The MF were proton-transporting ATP synthase activity (GO:0046933), NADH dehydrogenase (ubiquinone) activity (GO:0008137) and, electron carrier activity (GO:0009055), also associated to the muscle energy metabolism.

Seventeen KEGG pathways were identified by DAVID (Fig. 4, Additional file 4: Table S3) contrasting with 21 showed in WebGestalt enrichment analysis (FDR 5%, Additional file 5: Table S4). All 17 pathways found by DAVID also appeared in WebGestalt analysis. The four different KEGG pathways identified by the second program (Table 3) were Arginine biosynthesis (bta00220), Phenylalanine, tyrosine and tryptophan biosynthesis (bta00400), Butanoate metabolism (bta00650) and, Lysine degradation (bta00310). Among the common pathways, we can highlight Fatty acid metabolism (bta01212), Oxidative phosphorylation (bta00190) and Citrate cycle (TCA cycle) (bta00020) associated to the muscle energy metabolism; and PPAR signaling pathway (bta03320), associated to the muscle lipid metabolism. And some pathways related to muscle degradation and oxidative stress diseases, such as Parkinson’s disease (bta05012) and Alzheimer’s disease (bta05010).

**Table 3:** KEGG Pathways (FDR < 0.05) unique identified by WebGestalt 2017 from the gene list of the Salmon module

KEGG Pathway ID^a^	Description	N Gene^b^	FDR^c^	Gene names
bta00220	Arginine biosynthesis	3	9.41E-03	*GOT1, GOT2, GPT2*
bta00400	Phenylalanine, tyrosine and tryptophan biosynthesis	2	1.75E-02	*GOT1, GOT2*
bta00650	Butanoate metabolism	3	2.70E-02	*HADHA, ACAT1, HADH*
bta00310	Lysine degradation	4	3.51E-02	*HADHA, DLST, ACAT1, HADH*

^a^KEGG Pathway Identification (ID); ^b^Number of genes; ^c^Adjusted p-value for a false discovery rate (FDR) of 5%

At last, the Ivory and Light Yellow modules, positively correlated to the BFT (*p*-value< 0.1), presented 17 co-expressed genes and, 87 co-expressed genes respectively (Additional files 6 and 7: Tables S5 and S6, respectively). The genes within these modules were not significantly enriched by DAVID (FDR 5%) nor by WebGestalt (FDR 5%).

## Discussion

The commercial value of the bovine carcass is determined by the adequate development of muscle and adipose tissue. Therefore, an increase in muscle and fat masses in growing cattle is an important issue for farmers and beef industry. Studies involving *Bos indicus* animals have shown a lower propensity for subcutaneous fat deposition and *Longissimus* muscle area when compared to *Bos taurus* breeds [2, 3]. Compared to other Nelore studies, our phenotypic values for REA (mean value = 59.75 cm^2^) can be considered on the average, taking into account that studies in Nelore cattle show amounts varying from 40 to 100 cm^2^ [1, 2, 20, 21]. On the other hand, the BFT phenotypic values (mean value = 7.00 mm) were higher than that found in the literature for Nelore, with mean values ranging from 1.93 to 4.84 mm and, even higher than presented in some *Bos indicus* x *Bos taurus* crossbreed studies [1, 3, 20–22].

The variation in ribeye area and backfat thickness in Nelore cattle is harmful both for producers and the beef industry since these traits influence carcass yield and consequently, producer’s payment [3]. It’s well known that both environment and genetics contribute to the phenotypic variations within a population or in the same breed. So, to better understand the genetic and molecular influences behind these economically important traits, our goal was to construct co-expressed gene networks and identify putative candidate genes and metabolic pathways that regulate these traits, using the WGCNA tool and RNA sequencing data.

Forty-six gene clusters were constructed, between them, three were positively correlated (*p*-value< 0.1) to the BFT (Green Yellow, Ivory, and Light Yellow modules) and, one cluster was negatively correlated (*p*-value< 0.1) with REA (Salmon module). From the three positive correlated modules assigned to BFT, just the genes within the Green Yellow were significantly (FDR 5%) enriched by DAVID and WebGestalt.

The functional enrichment analysis for the co-expressed genes from the Green Yellow module demonstrated that they are mainly involved in inflammatory mechanisms and immune response. In previous work, Oliveira et al. [18], studying intramuscular fat deposition in this same Nelore cattle population also found co-expressed gene modules enriched for inflammatory response and immune system GO terms. Tao et al. [23] studying the muscle and adipose transcriptomic profile of Indigenous *x* Western Chinese pig breeds associated with growth performance and quality carcass traits – intramuscular fat, marbling, and loin muscle area –, identified genes related to immune response and inflammation. These authors identified highly expressed and up-regulated genes in the muscle tissue of Chinese Indigenous breeds (higher BFT content) enriched for immune response, mitochondria, Herpes simplex infection, Parkinson’s disease, and apoptosis GO terms, corroborating our findings.

The backfat, not only, is important in the industrialization process acting as a thermal insulator during carcass cooling but is an indispensable source of energy in the animal’s body, carrying fat-soluble vitamins and essential fatty acids [2, 3, 24, 25]. This trait also is representative of the total body fat in the carcass. According to Schröder and Staufenbiel [26], an increase of just 1 mm in the BFT reflects in approximately 5 kg of the total body fat content in bovine.

The increase in total body fat is defined by an adipose tissue expansion together with adipocyte hypertrophy [27]. The fat deposition is a consequence of the balance between energy intake and expenditure. The excess of nutrients and energy can lead to an increase in fat accumulation – also called obesity in human and mouse studies – and, consequentially activate inflammatory and stress responses. So, this inflammatory process created by the increase in fat can disrupt systemic metabolic homeostasis and inhibit insulin receptor signaling, involving immune cells and immune response pathways. Although the adipose tissue is the primary source of inflammation induced by excess fat accumulation, it is sufficient to activate inflammatory signaling pathways and increase the amount of pro-inflammatory immune cells in other tissues, like skeletal muscle, liver, pancreas, and brain [28–32]. The molecular mechanisms behind fat deposition in bovine are still unclear, so studying the lipid metabolism from another mammalian species can clarify our knowledge about the differences in backfat in this cattle population.

In our preview study, with the same Nelore population [64], we identified biological processes related to the immune response in differentially expressed genes assigned to the BFT trait, similarly to the results found herein. Interestingly, the *RSAD2* gene (Radical domain of S-adenosyl methionine containing 2) was down-regulated in the group with lower GEBV values for BFT in our previous study and, herein it was identified in Green Yellow module, which was positively correlated to BFT (*p*-value< 0.1).

*RSAD2* is an interferon-regulated gene associated with innate immune response during viral infections [33, 34]. Additionally, this gene participates in lipid biosynthesis and the modulation of lipid droplet contents [35, 36]. Dogan et al. [36], working with obese-induced mouse detected higher expression of *RSAD2* in animals with lower fat amounts, showing that this gene controls lipid droplets formation, but it is the *RSAD2* impairment that drives fat accumulation. These authors further associated this gene with endoplasmic reticulum (ER) stress and the activation of inflammatory mechanisms in obese animals. According to Warfel et al. [31] and Ramos-Lopez et al. [37], one of the main contributors to the activation of inflammatory pathways and immune response during obesity is the metabolic stress of organelles, such as ER and mitochondria.

Like *RSAD2*, the *EIF2AK2* (Eukaryotic translation initiation factor 2 alpha kinase 2; Alias *PRKR, PKR*) is a key inducer of inflammation, responsive to interferons (IFN) and, associated with ER stress and fat accumulation [37, 38]. In Green Yellow module, *EIF2AK2* was associated with inflammatory pathways, and immune response GO terms. This gene encodes a protein called PKR, a serine/threonine kinase, activated by double-stranded RNA, cytokines, stress signals, and IFN. The PKR protein can be activated by lipids and, participate in major inflammatory signaling events, such as JNK (c-Jun N-terminal kinase) activation during lipids exposition and ER stress [28, 38]. Nakamura et al. [28] hypothesized that the increase in PKR activity in obese states could be caused by an excess of energy and nutrient supply, representing an adaptive attempt to interact with synthetic pathways that would further accumulate energy. In our study, higher expression of the *EIF2AK2* gene was positively correlated with BFT, supporting the association of this gene with fat accumulation.

Another gene family identified in the Green Yellow module was poly (ADP-ribose) polymerases (PARP) represented by *PARP9, PARP10, PARP12,* and *PARP14.* PARPs activity is stimulated by excess fat, high-fat diet, aging, oxidative stress, DNA damage and, inflammation states. These enzymes are involved in lipid metabolism, mostly by controlling redox balance and NAD^+^ homeostasis in mitochondrial metabolism [39, 40]. According to Jokinen et al. [39], obesity can be characterized by low levels of NAD^+^ in the adipose tissue that can stimulate PARP activity. Mohamed et al. [41] examined the effect of high-fat diet in mouse skeletal muscle cultured cells (C2C12), and they found that oversupplied obese animals had their levels of *PARP2* increased together with reduced mitochondrial functions and NAD^+^ levels in the cultured muscle cells.

The NOD-like receptor signaling pathway (bta04621), the only one enriched by WebGestalt (Table 2), participates in the innate immune response and, can be activated by increased levels of glucose and free fatty acids, as well as reactive oxygen species derived from the inflammation state during obesity [27, 42]. Yin et al. [27] studying adipocytes metabolism from the adipose tissue of obese *x* non-obese women, verified an up-regulation of the NOD-like receptor signaling pathway in the adipocytes from the obese group. So, genes and metabolic pathways presented in the Green Yellow module, related to immune response and inflammatory processes, have an important paper in the lipid metabolism in mammals, demonstrating that there is a correlation between the increase in the expression of genes involved in these pathways and the BFT in cattle.

The other significantly enriched gene module, Salmon, showed a negative correlation (*r* = − 0.3, *p*-value< 0.1) with the ribeye area. REA is an important quality carcass trait related to the amount of meat, that is used as an indicator of cuts yield, the percentage of muscle, animal growth and carcass weight [1–3, 43].

Some of some GO terms and KEGG pathways identified here, like the fatty acid beta-oxidation (GO:0006635), mitochondrion (GO:0005739), mitochondrial proton-transporting ATP synthase complex (GO:0005753), Citrate cycle (TCA cycle) (bta00020) and, Oxidative phosphorylation (bta00190) are part of the complex cascade of events that occur in the skeletal muscle for generate energy. Muscles have an essential role in energy metabolism, the regulation of skeletal muscle metabolism involves multiple pathways and different molecules committed in the uptake and storage of energy. The glucose and fatty acid metabolism are the major sources of energy in this tissue. Their energy demand is mainly fulfilled by phosphocreatine and ATP produced during glucose and fatty acid oxidation [44]. In the glucose metabolism, glucose delivered by blood enters the myocyte, and its oxidation generates energy by phosphorylation. In the fatty acid metabolism, the primary source of energy for the muscle is the non-esterified fatty acids (NEFA) derived from circulation and, from lipolysis of triacylglycerols (TG) located mostly in the adipose tissue or, accumulated in the muscle (intramuscular TG). Once in the cytosol, NEFA are esterified to long-chain acyl CoA that is destined for mitochondrial beta-oxidation and, subsequently enter the TCA cycle generating ATP [45, 46]. Cesar et al. [47] and Oliveira et al. [18], studying fat-related traits in this Nelore population, also identified energy metabolism pathways in the skeletal muscle of the animals.

Marrades et al. [48], investigating two groups of subjects (lean versus obese) high feed diet consuming, identified *ACADM* gene (Medium chain acyl-CoA dehydrogenase) in the Fatty acid β-oxidation pathway and, *SUCLG2* (β subunit succinate-CoA ligase, GDP-forming) in the TCA cycle pathway, down-regulated in the subcutaneous adipose tissue of obese subjects. Likewise, Jeong et al. [49] identified the TCA Cycle and Fatty Acid Oxidation genes in the *Longissimus* muscle of Korean bulls, following castration. Their enrichment analysis also demonstrated *CPT1B* (Carnitine palmitoyltransferase IB) and Hydroxyacyl-CoA dehydrogenase (HADH) genes assigned to Fatty oxidation pathway; and, Succinate-CoA ligase [GDP-forming] (SUCLG), and Isocitrate dehydrogenase (IDH) gene families in the TCA Cycle pathway. Also, they found some ATP (Adenosine triphosphatase), NDUF (NADH dehydrogenase [ubiquinone]) and COX (Cytochrome c oxidase) genes present in the Oxidative phosphorylation pathway, similar to our findings.

Three HADH genes appeared in Salmon module enrichment analysis, *HADH* (Hydroxyacyl-CoA dehydrogenase), *HADHA* (Hydroxyacyl-CoA Dehydrogenase Trifunctional Multienzyme Complex Subunit Alpha) and, *HADHB* (Hydroxyacyl-CoA Dehydrogenase Trifunctional Multienzyme Complex Subunit Beta). Together they were identified in ten KEGG pathways, including two of the WebGestalt analysis (Table 3 and Additional file 4: Table S3). Costa et al. [50], studying the fatty acid profile of the *Longissimus* muscle of a cattle population, found *HADHA* gene associated with several terms related to the fatty acid metabolism, like oxidation of lipid and, accumulation of specific fatty acids. Furthermore, HADH enzymes are crucial in the mitochondrial beta-oxidation, participating in the two final steps of this process [51]. According to Zhang et al. [45] and Xu et al. [51], an up-regulation of the mitochondrial beta-oxidation process leads to a lower body fat content due to diminishing TG content. Maybe this could explain why some fatty acid metabolism terms and pathways appeared associated with REA, in our skeletal muscle study.

Adjacent to the pathways and terms associated with energy metabolism, we also found some associated with the muscle lipid metabolism, like the PPAR signaling pathway (bta03320). The PPAR pathway is responsible for the regulation of adipocyte tissue development, adipogenic differentiation and, lipogenesis. The PPARs (peroxisome proliferator-activated receptors) are nuclear receptors that take part in a number of biological processes, like skeletal muscle lipid oxidation, inflammation, mitochondrial respiration, energy homeostasis and, thermogenesis [52–54]. Several studies identified *PPAR* genes associated with fat traits in cattle [18, 47, 53–55].

Huang et al. [54] analyzing the transcriptomic profile of the subcutaneous adipose tissue from Wagyu and Holstein cattle confirmed the importance of the PPAR signaling pathway as a key regulator of lipid metabolism in bovine. These authors also found acetyl-CoA acyltransferase 1 (*ACAT1*) and acyl-CoA synthetase long-chain family member 1 (*ACSL1*) genes up-regulated in the BFT of Wagyu cattle. Here, the *ACAT1* and *ACSL1* genes were found in a gene module negatively associated with REA. These genes not only were verified in the PPAR signaling pathway; *ACAT1* was identified in four GO terms and nine pathways (Additional file 4: Table S3), two of them only by WebGestalt (Table 3) – Butanoate metabolism (bta00650) and Lysine degradation (bta00310). The *ACSL1* was found in the mitochondrion (GO:0005739) and, four KEGG pathways (Additional file 4: Table S3).

*ACAT1* is a fatty acid deposition gene that catalyzes the conversion of cholesterol to cholesteryl esters [56–58]. Yue et al. [58] selected the *ACAT1* as a candidate gene to study adipogenesis in bovine adipose-derived mesenchymal stem cells. Like *ACAT1*, *ACSL1* is crucial for the lipid metabolism, contributing to fatty acid biosynthesis, transport, storage and degradation; and, taking part in the mitochondrial beta-oxidation process [54, 59]. Zhao et al. [60] evaluating different tissues of Qinchuan cattle verified that *ACSL1* mRNA was highly expressed in the skeletal muscle (*Longissimus thoracis*) and subcutaneous fat, affirming that this gene may contribute to the determination of fatty acid composition in bovine skeletal muscle. Recently, Poleti et al. [61] found ACSL1 protein as differentially abundant when studying the intramuscular fat deposition in the LD muscle of this Nelore population.

Another noteworthy gene verified in the PPAR pathway is *SLC27A6* (Solute Carrier Family 27 Member 6), part of the solute carrier superfamily (SLC). The solute carrier family 27A (SLC27A) is a group of molecules ubiquitously expressed, involved in the lipid metabolism by transporting fatty acid proteins [62, 63]. According to Melo et al. [63], despite the SLC27A family is essential for body lipid distribution, the *SLC27A1* was found more expressed in the skeletal muscle than adipose tissue, suggesting that this gene has a critical role in the absorption and storage of fatty acids by the muscle.

Beside the *SLC27A1*, we also identified other SLC members in the Salmon module – *SLC25A3, SLC25A4, SLC25A11, SLC25A12* and, *SLC26A9* – associated to three cellular components and two oxidative stress diseases pathways (Additional file 4: Table S3). In previous work with this Nelore population, we have already found SLC genes more expressed in the group with the lowest GEBV for REA [64]. Junior et al. [1], considered *SLC38A1* and *2* as candidate genes for muscle growth associated with REA, in a GWAS study with Nelore cattle. In another way, Costa et al. [50] identified the *SLC37A4* involved in relevant lipid metabolism biological functions, such as the concentration of lipid, metabolism of triacylglycerol and homeostasis of cholesterol, when studying the bovine *Longissimus* muscle. Thus, the enrichment analysis of the Salmon module demonstrates the complex regulation of skeletal muscle energy and lipid metabolism, helping us to understand molecular insights occurring in the bovine LD muscle that can influence REA.

## Conclusions

With the construction of co-expressed gene modules, we verified that inflammation and immune response pathways and biological processes could modulate the BFT in Nelore cattle. *RSAD2, EIF2AK2*, and *PARP* genes could be considered as putative candidate genes for BFT trait. For REA, we found that energy and lipid metabolism, mainly the fatty acid metabolism, were the major pathways regulating this trait in this cattle population. We highlight *ACAT1* and *ACSL1* as putative candidate genes, associated with the energy and lipid metabolism in the skeletal muscle. These results allow us a better comprehension of the molecular mechanisms that are behind these economically important traits, that lead to muscle and fat deposition in bovine.

## Methods

### Animals and phenotypes

A total of 385 Nelore steers descending 34 unrelated bulls, representing the main pedigree lineages of Brazilian Nelore cattle, raised between 2009 to 2011, were used in this study. All animals were raised in the same nutritional and handling conditions and, finished in feedlot. More details were provided in [19].

The animals were slaughtered at an average age of 25 months in a commercial abattoir located in Bariri (São Paulo, Brazil) under Federal Inspection Service (SIF) supervision and, Brazilian Ministry of Agriculture, Livestock and Food Supply (MAPA) regularization. As mentioned in our previous research [64], 5 g of the *Longissimus dorsi* (LD) muscle (12th-13th ribs) was collected from the right side of the carcasses at the time of slaughter and stored in liquid nitrogen until RNA-Sequencing analyses. A sample of the LD muscle (10th-13th ribs) was excised at 24 h after slaughter from the left side of the carcasses and transported to the Embrapa Pecuária Sudeste Laboratory (São Carlos, São Paulo, Brazil) to measure REA and BFT. The REA was dimensioned with a grid of points (values presented in cm^2^) and, the BFT was measured with a graduated ruler (values shown in mm). All the experimental procedures were approved by the Institutional Animal Care and Use Committee Guidelines from Embrapa (approval code CEUA 01/2013).

To the co-expression network analysis, we selected 48 animals with contrasting genomic estimated breeding values (GEBV, Additional file 1: Table S1) from the total population of 385 animals, where 12 represents the higher values and 12 the lowest values of REA (mean and SD values = 3.14 ± 0.56 and − 2.94 ± 0.59) and BFT (mean and SD values = 1.24 ± 0.28 and − 0.91 ± 0.12), respectively. After that, the lists of the animals were combined, and the repeated ones were removed, totaling 43 animals. The GEBV were calculated by GenSel program [65], based in the SNP marker information, obtained by the BovineHD 770 k BeadChip (Infinium BeadChip, Illumina, CA, USA), as described in [66]. The a priori genetic and residual variance values were obtained from the Bayes C analysis where the genetic and a priori residual variance was equal to 1 [67]. A new Bayes C analysis was performed with the previous values for genetic and residual variances to estimate the GEBV values for each animal. Correlation analysis between the phenotypic values of REA and BFT *x* the GEBV for these traits and, the GEBV for REA *x* GEBV for BFT were performed in the R program.

### RNA-sequencing

- *RNA extraction:* Total RNA was extracted from approximately 100 mg of muscle tissue (*n* = 43) using Trizol reagent (Life Technologies, Carlsbad, CA, USA), according to the manufacturer’s instructions. The RNA integrity was verified using Bioanalyzer 2100 (Agilent, Santa Clara, CA, USA). All the samples presented an RNA Integrity Number (RIN) greater than 7.
- *Library preparation:* A total of 2 μg of total RNA of each sample was used for library preparation following the TruSeq RNA Sample Preparation kit v2 guide (Illumina, San Diego, CA, USA) protocol. Libraries were quantified utilizing quantitative PCR with the KAPA Library Quantification kit (KAPA Biosystems, Foster City, CA, USA), and libraries mean size was estimated by the Bioanalyzer 2100.
- *Sequencing, quality control and alignment:* Samples were diluted for the same concentration and grouped in pools. The sequencing flowcell lanes were clustered with the TruSeq PE Cluster kit v3-cBot-HS (Illumina, San Diego, CA, USA) and then, sequenced using HiSeq2500 ultra-high-throughput sequencing system (Illumina, San Diego, CA, USA) with the TruSeq SBS kit v3-HS. A more detailed description can be found in [47]. SeqClean software (https://sourceforge.net/projects/seqclean/files/) was employed to remove the adapters sequences used in the library preparation step, and low-complexity reads. For the quality control, FastQC version 0.10.1 software (http://www.bioinformatics.babraham.ac.uk/projects/fastqc/) was applied. TopHat version 2.1.0 software [68] was used to map the read alignment against the reference genome *Bos taurus UMD3.1* (http://www.ensembl.org/Bos_taurus/Info/Index/). Lastly, read counts for all annotated genes were calculated adopting HTSeq software version 0.6.1 (https://htseq.readthedocs.io/en/release_0.10.0/) [69]. Only read sequences that uniquely align to know chromosomes were used in this study.

### Co-expression network analysis

To perform the co-expression network analysis, we used the WGCNA (Weighted Correlation Network Analysis) package from R [8], with RNA-Sequencing data (*n* = 43) with their counts normalized by transcript per million (TPM). After the data input, a cleaning and preprocessing step were done to remove outlier samples and genes with excessive numbers of missing entries. So, from a list of 15,631 genes inputted, 14,529 ones were used to construct gene networks with the “blockwiseModules” function.

First, a matrix of similarity was constructed by calculating Pearson correlations, to measure the similarity between the gene expression profiles of all the samples. Then, the similarity matrix was transformed into an adjacency matrix (*A*) raised to a β exponent (soft threshold) based on the free-scale topology criterion. In this study, the β parameter was equal to 6, and the free-scale topology was R^2^ = 0.80. The topological overlap matrix (TOM) was used to define modules based on dissimilarity (1-TOM). The minimum and maximum module size (genes per module) were five and 12,000, respectively. Modules were merged based on the dissimilarity between their eigengenes, which is the first principal component of each module and, represents the gene expression profile within the module [8]. For modules grouping, was used a threshold of 0.25 corresponding to a correlation of 0.75. Finally, for each gene module was assigned a color, genes not assembled to any modules were grouped in the Grey module.

Module-trait associations were estimated using the correlation between the module eigengene (ME) and the GEBV values of REA and BFT, allowing the identification of modules highly correlated with the interest traits. Genes of modules with significant module-trait associations (*p*-value< 0.1), for at least one trait, were assigned for functional enrichment analysis.

### Functional enrichment analysis

An Overrepresentation Enrichment Analysis (ORA) was performed by DAVID (Database for Annotation, Visualization and Integrated Discovery) version 6.8 [70]; and, WebGestalt 2017 (WEB-based GEne SeT AnaLysis Toolkit) [71]. DAVID software revealed all Gene Ontology (GO) terms (BP, CC, and MF) and KEGG pathways of the co-expressed genes with a False Discovery Rate (FDR) of 5%. WebGestalt was used to find additional relevant KEGG pathways (FDR 5%) that may not appear in DAVID analysis, to better understand the biological mechanisms involved in each trait studied. The *Bos taurus* genome was used as background for both analyses.

## Additional files

Additional file 1: **Table S1.** Test of means (t-test) of backfat thickness (BFT) and ribeye area (REA) between groups with High (H) and Low (L) genomic estimated breeding values (GEBV) for REA and BFT in the *Longissimus dorsi* muscle of Nelore steers. (XLS 35 kb)

Additional file 2: **Figure S1.** Heatmap plot of the gene network using a subset of 400 genes. The heatmap plot depicts the Topological Overlap Matrix (TOM) among a subset of 400 genes from the analysis. Each row and column represents a single gene. The light colors represent the low overlap between modules, progressively darker red color represents higher overlap. Darker color blocks along the diagonal represent gene modules. The gene dendrogram and module assignment are shown above and along the left side of the graph. (PNG 107 kb)

Additional file 3: **Table S2.** Functional enrichment analysis from the gene list of the Green Yellow module, performed by DAVID v6.8 (FDR < 0.05). The table contains the Gene Ontology category, identification and description, *p*-value adjusted for a false discovery rate of 5%, nominal *p*-value, number of genes and gene names for each category. (XLS 30 kb)

Additional file 4: **Table S3.** Functional enrichment analysis from the gene list of the Salmon module, performed by DAVID v6.8 (FDR < 0.05). The table contains the Gene Ontology category, identification and description, *p*-value adjusted for a false discovery rate of 5%, nominal *p*-value, number of genes and gene names for each category. (XLS 38 kb)

Additional file 5: **Table S4.** KEGG Pathways (FDR < 0.05) identified by WebGestalt 2017 from the gene list of the Salmon module. The table contains the KEGG Pathway identification, description, number of genes and gene names for each pathway. (XLS 31 kb)

Additional file 6: **Table S5.** Gene list from the Ivory module. The table contains the Ensembl gene identification, gene symbol and gene description of the entire list of genes from the Ivory module. (XLS 28 kb)

Additional file 7: **Table S6.** Gene list from the Light Yellow module. The table contains the Ensembl gene identification, gene symbol and gene description of the entire list of genes from the Light Yellow module. (XLS 38 kb)

## Abbreviations

BFT: Backfat thickness
BP: Biological process
CC: Cellular Component
DAVID: Database for Annotation, Visualization and Integrated Discovery
ER: Endoplasmic reticulum
FDR: False discovery rate
GEBV: Genomic estimated breeding value
GO: Gene Ontology
IFN: Interferons
KEGG: Kyoto Encyclopedia of Genes and Genomes
LD: *Longissimus dorsi*
ME: Module eigengene
MF: Molecular function
REA: Ribeye area
TPM: Transcript per million
WebGestalt: WEB-based GEne SeT AnaLysis Toolkit
WGCNA: Weighted Correlation Network Analysis
