# Supplementary figures and images for: Gene co-expression networks associated with carcass traits reveal new pathways for muscle and fat deposition in Nelore cattle
Source: BMC Genomics. 2019 Jan 10;20:32. doi: 10.1186/s12864-018-5345-y (PMC6329100; doi:10.1186/s12864-018-5345-y)

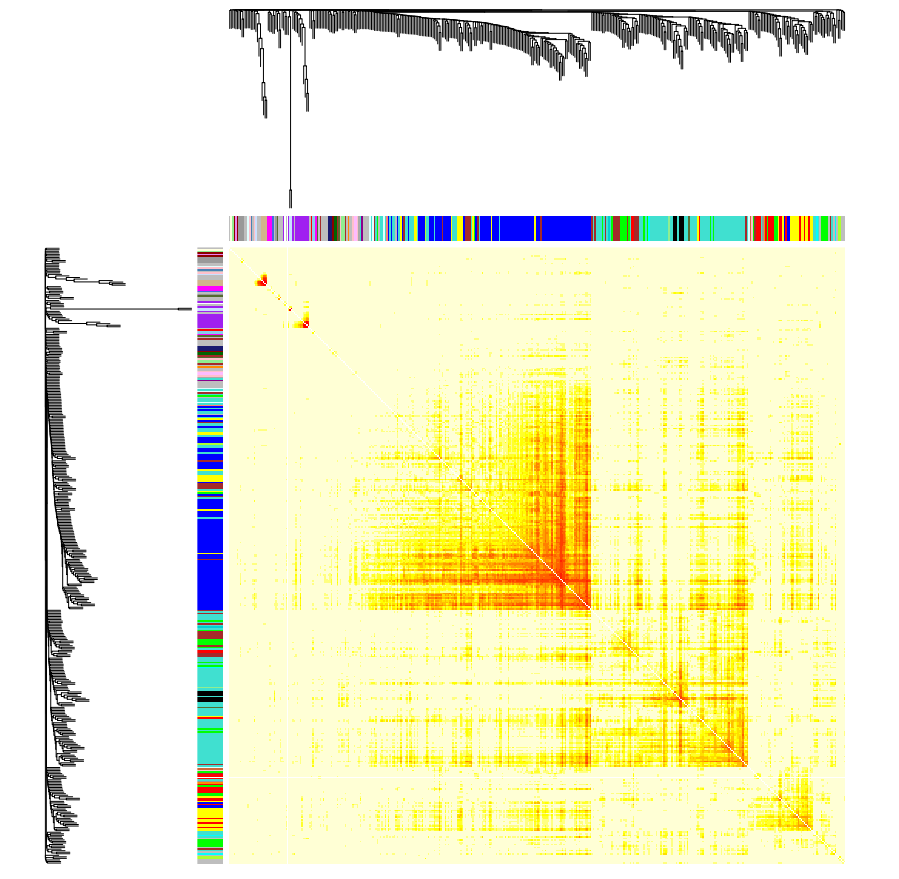

Supplement: Supplementary file 2 — Figure S1. Heatmap plot of the gene network using a subset of 400 genes. The heatmap plot depicts the Topological Overlap Matrix (TOM) among a subset of 400 genes from the analysis. Each row and column represents a single gene. The light colors represent the low overlap between modules, progressively darker red color represents higher overlap. Darker color blocks along the diagonal represent gene modules. The gene dendrogram and module assignment are shown above and along the left side of the graph. (PNG 107 kb) [file 12864_2018_5345_MOESM2_ESM.png]
